# Coronary artery fistula between single right coronary artery and right pulmonary artery: a case report and literature review

**DOI:** 10.1186/s12872-015-0166-2

**Published:** 2015-12-16

**Authors:** Li-Jian Xie, Li Zhang, Ting-Ting Xiao, Jie Shen

**Affiliations:** Department of Cardiology, Shanghai Children’s Hospital, Shanghai Jiaotong University, Postal address: No. 355 Luding Road, Postcode: 200062 Shanghai, China

**Keywords:** Coronary artery fistula, Single coronary artery, Angiography

## Abstract

**Background:**

Coronary artery fistula and single coronary artery are two different rare congenital anomalies. The cases with co-existed the two anomalies are more rare. To the best of our knowledge with literature review, the coronary artery fistula between single right coronary artery and right pulmonary artery has not been previously reported.

**Case presentation:**

In the present article, we report a Chinese patient (a 8-month-old male) who presented cyanosis when cried and heart murmur. The cardiac angiography confirmed coronary artery fistula between single coronary artery arising from the right aortic sinus and right pulmonary artery. Furthermore, the right pulmonary artery was interrupted with main pulmonary artery and the pulmonary blood supplied by single right coronary artery. Following the surgical procedure, the anomalous fistula vessel was cut and sutured. The right pulmonary artery was reconstructed to connect with main pulmonary artery. The patient had an uneventful postoperative course and discharged. Then we reviewed the related literature with Medline and Pubmed databases for further details.

**Conclusion:**

We believe our patient is the very particular case about coronary artery fistula combined with single coronary artery, and it is first reported with our literature review. As other coronary anomalies, coronary or aortic root angiography is the gold standard method for the diagnosis. Furthermore, early surgery is an optimal treatment in our case.

**Electronic supplementary material:**

The online version of this article (doi:10.1186/s12872-015-0166-2) contains supplementary material, which is available to authorized users.

## Background

Coronary artery fistula (CAF) has been described as a direct connection between a coronary artery and one of the cardiac chambers, large vessels or other vascular structures [1]. This abnormality accounts for 0.27–0.40 % of all congenital cardiac defects [2]. Congenital anomalies of the coronary arteries occur in 0.4–2 % of the population [3]. It is well known that certain coronary artery anomalies, including single coronary artery (SCA), Bland-White-Garland syndrome, coronary aneurysm, and CAF, can be associated with fatal complications [4].

However, SCA combined with CAF is very rare and easily misdiagnosed. Here we report a rare case of a SCA arising from the right aortic sinus associated with CAF with right pulmonary artery (RPA). Furthermore, RPA is interrupted with main pulmonary artery (MPA) and directly connected with SCA, combined with patent duct arteriosus (PDA) and atrial septal defect (ASD). The anomalies were successfully corrected with operation in our hospital. The case is first reported as we reviewed the literature with Medline and Pubmed databases.

## Case presentation

A 8-month-old Chinese boy (Han race) was admitted with heart murmur and cyanosis when cried or exercised. The boy was the mother’s first child from her first pregnancy. He was breech delivered and his birth weight was 3.05 kg. His Apgar scores were not known. His development was nearly normal and the body weight was 8.0 kg until 8 month old. Physical examination found a grade 3/6 continuous murmur in the second to fourth intercostal space at the left sternal border. The electrocardiogram showed sinus rhythm, right atrium and ventricle hypertrophy. The chest X-ray showed heart shade enlargement, cardiothoracic ratio was 66 %. Transthoracic echocardiography revealed SCA arising from the right aortic sinus and CAF with RPA, RPA was interrupted with MPA, ASD and PDA.

Heart catheterization demonstrated left pulmonary artery (LPA) capillary wedge pressure of 13/2 mm Hg (mean 9), MPA pressure 80/35 mm Hg (mean 55), right ventricular (RV) pressure 88/2 mm Hg (mean 12), left ventricular (LV) pressure 81/5 mm Hg (mean 38), descending aorta artery pressure 74/41 mm Hg (mean 58). Oxygen saturation was 86 % in aorta artery, 62 % in superior vena cava and 64 % in MPA. The ratio of Qp/Qs was 1.09. The total pulmonary resistance was 4.2 wood and the pulmonary arteriolar resistance was 3.6 wood. Selective aortic root angiography showed a SCA arising from the right aortic sinus and a dilated and tortuous fistula between SCA and RPA (Fig. 1.1–2). Furthermore, the blood flow of RPA was supplied by SCA arising from the right aortic sinus (Fig. 1.1–2). Selective RV angiography showed the dilated MPA was connected with LPA and interrupted with RPA (Fig. 1.3). So we made a diagnosis of SCA arising from the right aortic sinus with a CAF draining into the RPA, pulmonary hypertension and surgical correction was recommended (Additional file 1, avi style).

**Fig. 1:**
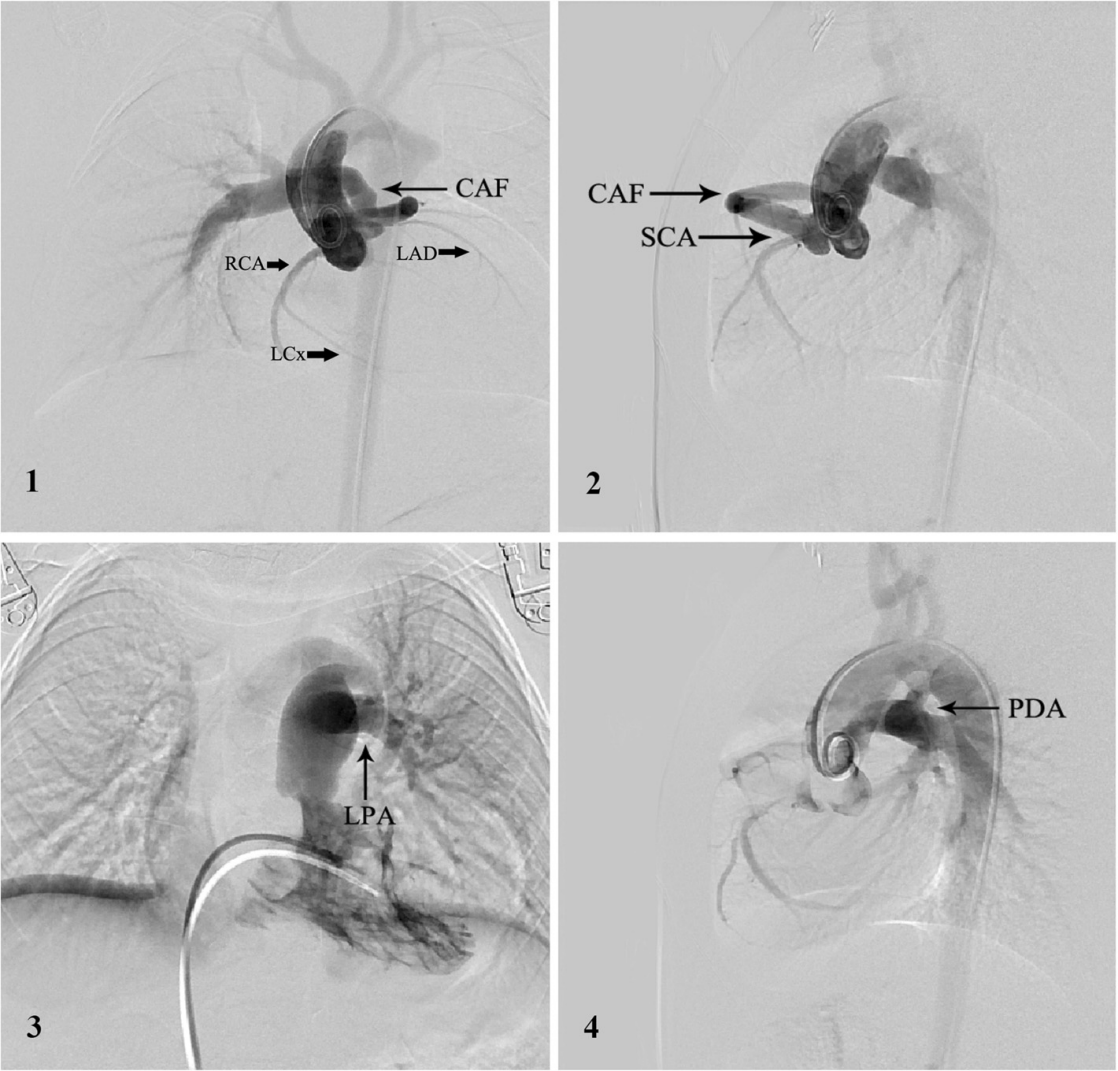
1 and 2: Selective aortic root angiography showed a right SCA arising from the right coronary sinus and a dilated and tortuous fistula between SCA and RPA, with the LCx and LAD arising separately from the common trunk, and, the blood flow of RPA was supplied by right SCA (A-P and lateral position). 3: Selective RV angiography showed the dilated MPA was connected with LPA and interrupted with RPA. 4: Selective aortic root angiography showed a vertical PDA between aortic arch and LPA

**Fig. 2:**
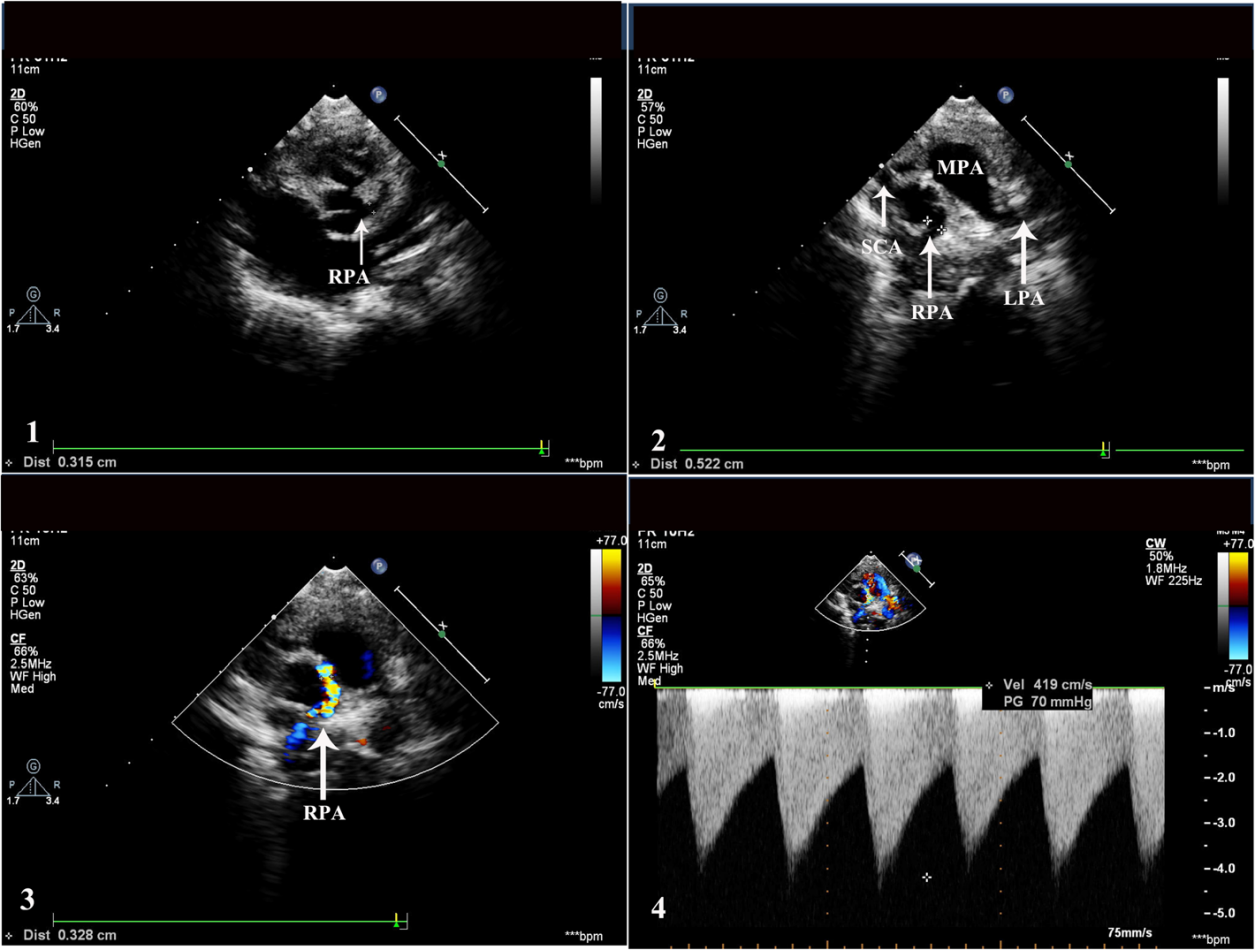
1 and 2: The reconstructive RPA originated from MPA, SCA arising from right aortic sinus in short axis view of echocardiography. 3: The color flow Doppler showed turbulent flow in RPA. 4: Continuous wave Doppler showed RPA blood flow velocity (4.19 meter/sec). SCA: single coronary artery; MPA: main pulmonary artery; RPA: right pulmonary artery; LPA: left pulmonary artery; LCx: left circumflex coronary artery; LAD: left anterior descending coronary artery; RV: right ventricle; PDA: patent duct arteriosus

Following median sternotomy, the pericardium was opened. The right atrium and ventricle were enlarged and MPA was dilated. An isolated SCA was found arising from the right aortic sinus and a tortuous anomalous vessel that ran to the RPA was identified, also, RPA was interrupted with MPA and RPA blood flow was supplied by CAF. After the cardiopulmonary bypass established, first,arterial duct was separated and sutured. Then the anomalous fistula vessel connected between SCA and RPA was separated and cut. The fistula was continuously sutured by 6/0 Prolene line. The RPA and MPA vascular anastomosis was successfully made. At last, the right atrium was opened and ASD was closed with continuous suture. The total time of bypass was 67 min. The patient had an uneventful postoperative course and discharged. On follow-up echocardiography, performed 2 weeks, 1 and 3 months after surgical treatment, no residual fistulous communications were detected, however, the reconstructive RPA blood flow velocity was increased (4.19 meter/sec). Then, we follow up the RPA blood flow velocity and the percutaneous RPA balloon or stenting will be considered (Fig. 2).

An isolated SCA is defined when only one coronary artery arises from the aorta by a single coronary ostium, supplying the entire heart [5]. SCA is a very rarely encountered disease entity, and its reported incidence in adults is only from 0.024 % to 0.066 % [5, 6]. It is most commonly found as an isolated finding (60 %), but it has also been associated with other congenital heart disorders (40 %) with a higher mortality [7–11]. Several classifications have been suggested with the one proposed by Lipton et al. [5] being the most commonly accepted [6, 7].

Based on Lipton classifications [5], the first division was made between the right-type (R) and left-type (L) according to the site of origin of SCA. Next, the artery was designated as group I, II, or III depending on its anatomical course. Group I had an anatomical course of either a RCA or LCA. Group II anomalies arise from the proximal part of the normal RCA or LCA, and cross the base of the heart before assuming the normal position of the inherent coronary artery. Group III describes SCA originating from the right sinus of valsalva, the left anterior descending (LAD) and left circumflex (LCx) branch arise separately from a common trunk. According to this classification [5], our patient presented SCA arising from the right aortic sinus with LAD and LCx originated from SCA, so it was classified as R- III type. It is important to know the origin and distribution of Lcx, especially to know whether LCx crossing the RV outlet tract. The LCx crossing the RV outlet tract could affect the correction of combined anomalies such as RV outlet tract stenosis.

CAF is a rare condition of a direct communication between a CA and one of the cardiac chambers or vessels. Moreover, few cases of SCA combined with CAF have been reported so far. With the literature review, the SCA combined with CAF connected with atrium, ventricle and MPA were reported [2, 4, 12–25]. The CAF originated from the LCA more commonly than from the RCA and most of the CAF ended in the RV [4]. However, the SCA combined with CAF connected with RPA has never been reported before.

CAF is suspected when a continuous murmur with diastolic accentuation at the left sternal edge is heard on a routine clinical examination. Most patients with CAF remain asymptomatic, but elderly patients can present with exertional chest pain and dyspnea, fatigue, congestive heart failure, palpitations, or arrhythmias [26]. Most fistulas are small and hemodynamically inconsequential. However, some can be large and lead to preferential blood flow from coronary circulation to low-pressure pulmonary circulation, resulting in pulmonary hypertension and coronary-steal-related chronic myocardial ischemia [26]. The prognosis of individuals with an isolated SCA anomaly is uncertain; major adverse cardiac events occur in 15 % before the age of 40 years [7]. Despite an acute takeoff angle typically not observed in patients with SCA, as seen in other abnormalities, risk of sudden cardiac death is increased, likely because of high coronary flow, which may predispose to early atherosclerotic disease through endothelial injury [27]. Currently, no treatment guidelines or follow-up recommendations exist [28].

Selective angiography allows detection of SCA associated with CAF in most patients during childhood. In our case, the aortic root angiogram could show the origin and ending of fistula, so it avoid the risk of coronary artery (CA) spasm in the infant. On the other hand, a newer technology, like multidetector-Row computed tomography, is a noninvasive tool recommended by the American Heart Association Committee to evaluate suspected or known CAF [29, 30]. No consensus exists on the optimal management of CAFs. Management of these remains controversial, especially in asymptomatic patients. Various treatment modalities, like coil embolization, catheter-mediated stent occlusion, and surgical ligation are available [26]. Small CAFs in children tend to grow with age. If untreated, fistulas cause clinical symptoms in 19 % of patients aged younger than 20 years and in 63 % of older patients [31]. In such context, early surgical correction is indicated because of the high prevalence of late symptoms and complications, especially when the shunt is significant (Qp/Qs ratio > 1.5) [32].

Our case is the first right SCA with RCA-RPA fistulous communication with literature review, and also combined with PDA and ASD. Early surgical closure of the fistula and other cardiac anomalies correction were done because of the patient’s hypoxia and pulmonary hypertension. The case had an uneventful recovery and no serious complications.

## Conclusion

We believe that this is the first report of a pediatric case of right SCA with CAF ending to RPA. Its diagnosis is important because of the potential therapeutic implications. As with many other coronary anomalies, coronary or aortic root angiography is the gold standard method for the diagnosis. Furthermore, early surgery is an optimal treatment in our case.

### Ethics

The study was performed in accordance with the Declaration of Helsinki and was approved by the institutional ethical board of Shanghai Children’s Hospital.

### Consent

Written informed consent was obtained from parents for publication of this case report and any accompanying images. A copy of the written consent is available for reviewed by the Editor-in-Chief of this journal.

## Additional files

Additional file 1:
**Selective cardiac angiography.** (AVI 40968 kb)

## Abbreviations

ASD: atrial septal defect
CA: coronary artery
CAF: coronary artery fistula
LAD: left anterior descending
LCx: left circumflex
LPA: left pulmonary artery
LV: left ventricular
MPA: main pulmonary artery
PDA: patent duct arteriosus
RPA: right pulmonary artery
RV: right ventricular
SCA: single coronary artery
